# Misdiagnosis of a giant intrapelvic schwannoma: A case report

**DOI:** 10.3892/ol.2013.1634

**Published:** 2013-10-15

**Authors:** FAN ZOU, MIN DAI, BIN ZHANG, TAO NIE

**Affiliations:** Department of Orthopedics, The First Affiliated Hospital of Nanchang University, Nanchang, Jiangxi 330006, P.R. China

**Keywords:** misdiagnosis, ovarian teratoma, pelvic cavity, schwannoma

## Abstract

Presacral schwannomas are a rare disease. The current case report presents a case of giant schwannoma with severe abdominal pain, constipation and dysuresia. The patient was initially diagnosed with an ovarian teratoma, which is an extremely common disease. The pain pattern and accompanying symptoms were the major factors indicating a teratoma. Abdominal computed tomography (CT) scans were the main tools of differential diagnosis, but the unsharpness of CT often disturbs diagnosis. Diagnosis of the tumor was confirmed by histopathology and immunohistochemistry, revealing a benign presacral schwannoma. The patient underwent complete resection and recovered well, albeit with a large amount of blood loss. The tumor was 25×15×10 cm^3^ in size and in contact with the sacrum. The aim of the present study was to review the diagnostic techniques of careful radiological examination. A follow-up was performed 2 years following the surgery and the patient remained alive and a CT scan demonstrated no evidence of recurrence. However, the long term efficacy of this process requires continuous observations of the patient.

## Introduction

Schwannoma originating from the sacral nerve is a rare and slow growing tumor, which arises most commonly in the peripheral nervous system (1). Although several case reports (2–6) have previously described schwannoma arising in unusual sites, the pelvis is also a rare site for a primary schwannoma. In the current case study, the patient was mistakenly diagnosed with benign ovarian teratoma without characteristic symptoms. The results of the computed tomography (CT) scan conformed to an ovarian teratoma. A careful patient history and clinical examination are important for identifying illnesses, particularly tumors. Further diagnostic pathology may clarify the diagnosis. However, definitive preoperative diagnosis of schwannomas is of great significance to guide treatment. To the best of our knowledge, this is the first description of a misdiagnosis of presacral schwannoma as an ovarian teratoma, cured with surgery. Written informed consent was obtained from the patient.

## Case report

**Figure 1:**
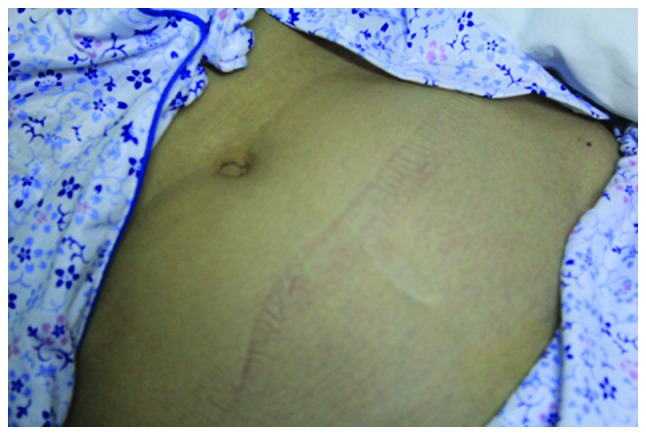
Preoperative local manifestation. The main symptoms were severe abdominal pain and apophysis.

**Figure 2:**
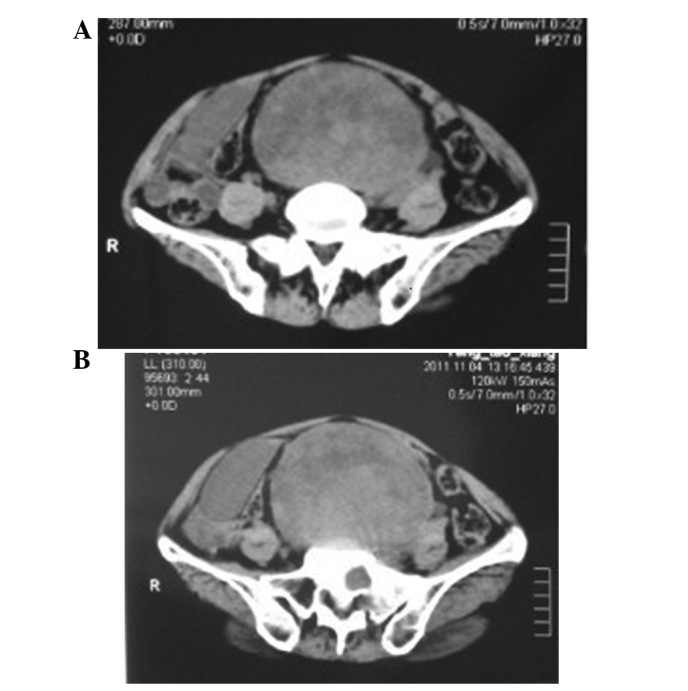
Preoperative CT scan observations. Screening CT showed a giant mass with uneven density originating from the sacral canal without bony destruction. There appeared to be several low-density areas within the tumor and the mass was closely attached to the (A) left ovary and (B) anterior wall of the sacrum. CT, computed tomography.

A 61-year-old female presented with low back ache of two months and severe abdominal pain and distention, constipation and dysuria of 2 weeks (Fig. 1). The patient was referred to the Gynecology Department for further investigation of a large uterine tumor and dilatation of the bilateral ureters detected by B-scan ultrasound, indicating a left adnexal mass. The patient had a previous history of cesarean section. Blood test results, including the levels of the tumor markers, carcinoembryonic antigen, α-fetoprotein (AFP) and carbohydrate antigens 19-9 and 12-5, were within normal limits. Screening CT showed a large mass with uneven density, 14×10×15 cm^3^ in size, located on the left ovary (Fig. 2A) and closely attached to the anterior wall of the sacrum without evident bony destruction (Fig. 2B). Enhanced CT scan revealed several irregular indurations of the nidus, however, no enlarged lymph nodes or distant metastases were found. Evidently, the bladder and uterus had become flattened by compression caused by the giant tumor. The CT demonstrated leftward hydronephrosis and thinned renal cortex also due to compression. The leftward ureter also appeared to be obstructed and dilated, further diagnosing a benign teratoma.

The surgery was completed through a collaboration between orthopedic surgeons and gynecologists. Firstly, surgery via an anterior approach was performed, ~5 cm in length. The patient’s uterus had become oblate and atrophic. The separation of the ovarian ligament was continued and the intestinal tube was pushed away with gauze. The tumor appeared grayish-white, elastic hard and smooth and appeared to be attached to the sacrum, 25×15×10 cm^3^ in size. The tumor was excised completely and tissue was extracted to perform pathological examination. The surgeon identified that the margin of the lesions had not invaded into the surrounding tissue and confirmed the tumor as benign. While removing the tumor, massive and rapid hemorrhage occurred as a result of injury to the presacral venous plexus. The surgery duration was 7.3 h and the estimated blood loss was 10,000 ml, with requirement of a blood transfusion of 28 units red blood cells suspension, 39 units fresh frozen plasma, 20 units platelets and 40 units cryoprecipitate, during the surgery. The patient experienced a challenging surgical procedure, but recovered completely following 10 days.

**Figure 3:**
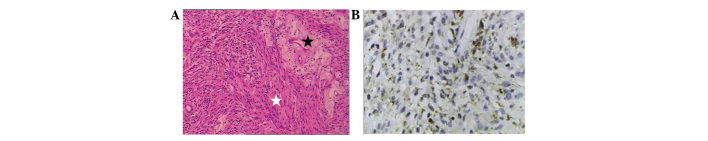
Histological observations. (A) Alternating Antoni A (white asterisk) and Antoni B (black asterisk) patterns (H&E; magnification ×100). (B) The tumor cells appeared locally positive for CD68 and negative for CD117, CD34 and desmin (S-P immunohistochemistry; magnification, ×200).

Inspection results by means of light microscopy revealed that the tumor was a benign schwannoma. The tumor was apparently spherical and circumscribed, with rich blood vessels. In addition, the cut surface was light yellow with a hard texture. Histologically, the tumor consisted of a small number of atypical and fibroblast-like cells with breezing borders. Sections of the cells were arranged in a scattered manner and oval vacuolated cell nuclei were observed. There were alternating hypercellular and hypocellular areas (Antoni A and Antoni B patterns). The hypercellular areas presented elongated spindle cells, along with nuclear palisading forming Verocay bodies; whereas the hypocellular areas presented loosely arranged neoplastic cells with thick-walled blood vessels (Fig. 3A). The tumor cells were rarely arranged into interlaced and circinate shapes and there was no observation of Verocay bodies. Immunohistochemical examination revealed that the tumor cells were locally positive for CD68, while negative for CD117, CD34 and desmin (Fig. 3B). Based on these characteristics, the tumor was finally diagnosed as giant schwannoma originating from sacrum. There has been no evidence of recurrence for 2 years since the surgery.

## Discussion

Schwannomas are benign neoplasms arising from the nerve sheath. Unusual locations, including the head, neck, peripheral nervous system, liver, pancreas, esophagus, stomach and peritoneum, have been previously reported (7–12). Schwannomas occurring in the pelvis are rare and account for only 1% of cases. Although the tumor arises from the peripheral nerve sheath, it rarely elicits any clinically detectable neurological deficits (13). Most often, detection is late and the tumors may be painless while growing to an extremely large size. The current case report showed that the patient exhibited severe and painful symptoms and presented with a giant abdominal mass with features of constipation and uroschesis. Compression of neurovascular structures explains the ability of the tumors to cause severe abdominal pain. However, schwannomas are rarely large enough to obstruct the lumen and ureter.

Ovarian teratomas are an extremely common type of abdominal tumor. Although fairly distinct in clinical and histological presentation, preoperative diagnosis of a schwannoma is not easy owing to a lack of distinguishing features on imaging studies, such as B-scan ultrasound or CT, between schwannomas and teratomas. MRI and CT are ideally suited to detect sacral pathology and delineate the soft tissue and bony components. The majority of benign ovarian teratomas without bony destroying present on CT scan (14). Nyapathy *et al*(15) previously reported a giant schwannoma arising from the sigmoid, with bone window axial CT sections of a large mass with erosions of the anterior cortex of the sacrum. In the present study, no bony destruction was found in the preoperative CT.

Previous studies have reported the misdiagnosis of schwannomas as psoas abscesses (16) and ovarian dermoid cysts (17), but rarely as ovarian teratomas occurring in the two genders and at all ages. Tumor markers, including AFP and CA-125, have contributed to the differential diagnosis between teratomas and schwannomas. However, the probability of a schwannoma while negative for these markers must be taken into consideration. It has been previously reported (18) that 7.5% of ovarian teratomas are positive for the expression of AFP and 22.6% for CA125. Therefore, AFP and CA125 are not the specific criterion for predicting ovarian teratomas. B-scan ultrasound revealing a leftward adnexal mass is likely to be an additional major cause of misdiagnosis. Preoperative CT also shows asymmetrical density of the tumor. In addition, specific symptoms, such as stomach ache, astriction and dysuria, were in accordance with the clinical manifestation of ovarian teratomas.

On the treatment side, surgical excision has been radically curative in a case of benign schwannoma and recurrence has been detected in few patients following incomplete removal. However, it must be noted that the pelvis is rich with blood vessels that favors hemorrhage during surgery. Tang *et al*(19) reported that 39.88% of the patients accepting sacral tumor resection exhibited blood loss of >3,000 ml. The authors results indicated that blood volume loss is affected mainly by tumor location, volume and blood supply. Hemorrhage is a serious intraoperative risk in cases where major vessels are situated near the tumor and there are currently several reports of unsuccessful tumor excision and intraoperative mortalities (20).

In summary, the present case report described a rare giant presacral schwannoma in the pelvic cavity, with notable observations of its clinical manifestations of constipation, dysuria and severe abdominal pain. Histological observations make a definitive diagnosis, but the preoperative clinical manifestations and details of the differential diagnosis were ignored. If fine-needle aspiration biopsy of the tumor had been offered, the misdiagnosis may have been avoided. The follow-up process has been accomplished and patients have recovered. The current report analyzed the clinical and imaging features of the tumor to highlight possible diagnostic and management methods.
